# Synthesis of Benzyl Acetate Catalyzed by Lipase Immobilized in Nontoxic Chitosan-Polyphosphate Beads

**DOI:** 10.3390/molecules22122165

**Published:** 2017-12-07

**Authors:** Ana D. Q. Melo, Francisco F. M. Silva, José C. S. dos Santos, Roberto Fernández-Lafuente, Telma L. G. Lemos, Francisco A. Dias Filho

**Affiliations:** 1 Instituto Federal de Educação, Ciência e Tecnologia do Ceará, Rod. Pres. Juscelino Kubitschek, Boa Viagem CEP 63870-000, Ceará, Brazil; 2 Instituto Federal de Educação, Ciência e Tecnologia do Rio Grande do Norte, RN 233, Km-02, Nº 999, Bairro Chapada do Apodi, Apodi CEP 59700-000, Rio Grande do Norte, Brazil; fmsilva1986@yahoo.com.br; 3 Instituto de Engenharias e Desenvolvimento Sustentável, Universidade da Integração Internacional da Lusofonia Afro-Brasileira, Redenção CEP 62785-000, Ceará, Brazil; jscleiton@gmail.com; 4 Department of Biocatalysis, ICP-CSIC, Campus UAM-CSIC, Cantoblanco, 28049 Madrid, Spain; 5 Departamento de Química Orgânica e Inorgânica da Universidade Federal do Ceará, Campus do Pici, Bloco 940, Fortaleza CEP 60455-760, Ceará, Brazil; tlemos@dqoi.ufc.br (T.L.G.L.); audisio@ufc.br (F.A.D.F.)

**Keywords:** chitosan, polyphosphate, microspheres, immobilization, lipase, CALB

## Abstract

Enzymes serve as biocatalysts for innumerable important reactions, however, their application has limitations, which can in many cases be overcome by using appropriate immobilization strategies. Here, a new support for immobilizing enzymes is proposed. This hybrid organic-inorganic support is composed of chitosan—a natural, nontoxic, biodegradable, and edible biopolymer—and sodium polyphosphate as the inorganic component. Lipase B from *Candida antarctica* (CALB) was immobilized on microspheres by encapsulation using these polymers. The characterization of the composites (by infrared spectroscopy, thermogravimetric analysis, and confocal Raman microscopy) confirmed the hybrid nature of the support, whose external part consisted of polyphosphate and core was composed of chitosan. The immobilized enzyme had the following advantages: possibility of enzyme reuse, easy biocatalyst recovery, increased resistance to variations in temperature (activity declined from 60 °C and the enzyme was inactivated at 80 °C), and increased catalytic activity in the transesterification reactions. The encapsulated enzymes were utilized as biocatalysts for transesterification reactions to produce the compound responsible for the aroma of jasmine.

## 1. Introduction

The use of enzymes as industrial catalysts and in organic synthesis is often convenient because enzymes are very specific, selective and capable of exhibiting a very high activity compared to conventional catalysts [1]. However, the use of enzymes for industrial bio-catalysis has certain problems such as the high production cost because they are synthesized in small concentrations by cells, and their extraction and purification are expensive. This high price is coupled to their moderate stability under operational conditions, the low activity versus non-physiological substrates, the non-absolute specificity or selectivity on industrially relevant substrates, etc. Some of these drawbacks may be overcome by the development of appropriate enzyme immobilization techniques, which should maintain (or even increase) enzyme activity, modulate enzyme selectivity or specificity, improve resistance to inhibitors [2,3,4] and may even increase enzyme purity [5], together with allowing an easy recovery of the catalyst from the reaction medium, thus reducing the overall operational costs [6,7,8].

The world market for industrial enzymes is dominated by products containing non-immobilized enzymes. This is justified by problems arising from the cost of the supports used for immobilization and the cost of the immobilization process. Moreover, an inadequate enzyme immobilization may negatively affect enzyme performance [7].

Among enzymes, the use of lipases as biocatalysts has been gaining prominence in the recent years, since they operate in different types of solvents (ionic liquids, supercritical fluids, organic solvents, and the least contaminant aqueous media), with many different substrates and being able to catalyze a broad range of reactions [9,10,11]. The use of lipases as catalysts results in selective synthesis, with low to no production of undesirable byproducts and being environmentally safe [12]. Thus, lipases are an important class of enzymes in biological systems [13,14], being the most commonly used biocatalysts, with applications in esterification, transesterification, and hydrolysis processes for a variety of chemical compounds [15]. In other words, in organic syntheses, lipases occupy a pivotal position [16].

The number of immobilization techniques that aim to improve the stability, activity, selectivity and specificity of lipases as well as their resistance to inhibitors or denaturants, has been increasing over the past years [2,6,17,18,19,20]. There are many techniques for enzyme immobilization, all of them having advantages and disadvantages [21]. There are two great types of approaches to enzyme immobilization, namely, irreversible and reversible. The most common irreversible methods are covalent binding [22], entrapment in supports [23,24], cross-linking of the enzymes that were previously physically adsorbed on the supports and microencapsulation. The most common reversible method of immobilization is physical adsorption [25].

Immobilization is in many instances associated with a decrease in enzyme activity produced by slight distortions in the enzymes’ structure or diffusional limitations, although in some cases an increase in enzyme activity is achieved [3]. A higher activity of a given immobilized enzyme may be derived from a decrease in enzyme inhibition or distortion, and not necessarily from the production of a more active conformation of the enzyme [15]. Prevention of enzyme dissociation in multimeric enzymes may be another way of improving enzyme activity or stability, mainly when used under dissociation conditions [26]. The activity of an enzyme depends on its three-dimensional structure, and the immobilization step can lead to altered conformational rearrangements, with positive or negative effects on enzyme activity [8].

One of the preferred techniques for enzyme immobilization is using pre-existing supports [22]; however, the production of *ex novo* solid supports may also be of great interest. These *ex novo* solid supports may be synthesized from a mixture of polymers with opposite ionic charges that maximize the stability, both enzymatic and physical, of an enzyme. Anionic polysaccharides, such as alginate [27,28,29], form coacervates maintained via strong interactions or microspheres with cations (e.g., Ca^2+^) or cationic polysaccharides, such as chitosan [30,31,32]. These immobilization strategies may be almost ideal to stabilize multimeric enzymes because enzyme dissociation will be avoided [26].

Chitosan is a natural polymer which is produced from chitin [33]. Chitin is normally isolated from the exoskeletons of many species of insects and crustaceans [34]. Furthermore, chitosan is a biocompatible gel-forming cationic compound that can readily be prepared in different geometrical configurations, such as membranes, beads, nanoparticles by nano-immobilization [35], fibers, hollow fibers or sponges [15,36,37,38,39].

Sodium polyphosphate is an inorganic, polymeric, poly anionic electrolyte with high cation sequestration ability [40]. Initially, it was named sodium hexametaphosphate, and later, the nomenclature was modified because the widely used method for the production of this polymeric salt produces a mixture of linear polymers with different lengths. Sodium polyphosphate is still prominent as the only water-soluble phosphate polymer, and because it consists of anionic groups that are distributed throughout the polymeric chain, cations can interact with its anionic chain [41]. Beads formed by sodium polyphosphate and chitosan were used to immobilize the lipase from *Rhizopus cohnii* [42]. In this new report, the enzymes are trapped in a support prepared using a different technique (see Section 3). That way the enzymes were simultaneously trapped and ionically exchanged.

The use of these two polymers has interest because they are known to be non-toxic and biodegradable and chitosan is commonly used as a support for the immobilization of lipases [43,44,45], being the use of polyphosphate comparatively less extended [42]. Chitosan is already used in numerous applications, with the approval of national and international regulatory agencies, as means of immobilizing drugs, in the preparation of protective films for food, in the treatment of water among other applications [46].

Rattanaphra and Srinophakun used chitosan as a support to immobilize lipase in transesterification reactions of sunflower and *jatropha* oil with methanol, finding that the transformation of sunflower oil is improved [47]. Although the synthesis of this ester by conventional chemical methods is possible, such production methods have many disadvantages, such as high temperature, toxicity of reagents, use of corrosive catalysts, and low selectivity, and thus, increasing the possibility of the generation of by-products that compromise the purity or hinder the purification of the target product [48]. Moreover, these esters cannot be labeled as a green product.

The interaction between the cationic groups of chitosan and a phospholipid monolayer was utilized to produce a support for immobilization was reported by Krajewska [49,50]. In this paper, the manufacture of a phospholipid monolayer following the Langmuir film technique and a study of the sensitivity of the layer to the temperature and the pH values, demonstrated that not only electrostatic forces were involved in the formation of the layer, but also significant non-electrostatic contributions had to be accounted for.

Esters are chemicals of great economic importance in the food, pharmaceutical, and cosmetic industries [51]. Among the esters of short-chain carboxylic acids, benzyl acetate is of outstanding interest, with applications in different fields. The annual production of this compound is up to 10,000 tons. This ester can be obtained from natural sources, since it is present in plants, such as jasmine and gardenia; however, its direct extraction and purification are highly complex and expensive [52].

The objective of this study was to analyze the effects of the encapsulation of a model enzyme on a novel *ex novo* support formed of chitosan and polyphosphate. As the model enzyme, we had selected the lipase B from *Candida antarctica* (CALB), which is one of the most widely reported enzymes in the literature [53,54,55,56]. This biocatalyst was utilized to produce benzyl acetate via a transesterification reaction. In the kinetically controlled reactions, the maximum yields are transient (the product could be hydrolyzed by the enzyme), and therefore the maximum yields are determined by the properties of the biocatalyst [4].

## 2. Results and Discussion

### 2.1. Characterization of Lipase Immobilized on Polyphosphate and Chitosan Support (LPCS) Microspheres

#### 2.1.1. Infrared Spectroscopy

The degree of chitosan deacetylation was directly related to the increase in the number of cationic sites present in the polymer. This could be followed by monitoring the axial deformation band of the C=O amide bond at approximately 1656 cm^−1^.

**Figure 1:**
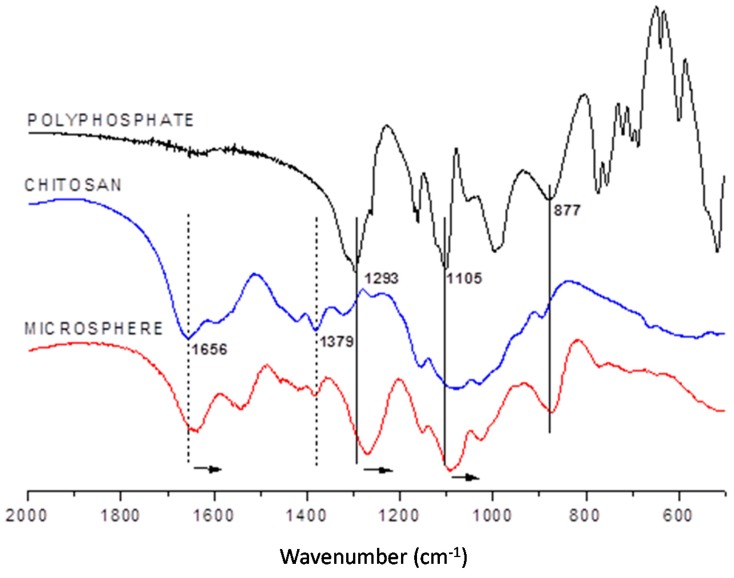
FT-IR spectra of polyphosphate, chitosan and the microspheres formed. The experiments were performed as described in Section 3.

The positions of the bands that characterized the center of the polyphosphate chain, the chain and the terminal atoms were displaced (they were ν_s_ (P-O-P), ν_s_ (terminal PO_3_), and ν_as_ (PO^2−^ middle of the chain) at 877, 1105, and 1293 cm^−1^, respectively. The displacement of these bands suggested that the center of the anionic chain and terminal sites of phosphate are involved in ion exchange with the cationic chain of chitosan. Figure 1 shows that the identity bands of each precursor polymer appeared in the spectral profile of the produced microspheres. This confirmed the hybrid (organic-inorganic) nature of the microspheres.

The new conformation of the material (microspheres of chitosan) after the interaction of the two macromolecules increased the energy. An evidence of these electrostatic interactions is the red shifts in the absorption bands [57].

#### 2.1.2. Thermogravimetric Analysis

**Figure 2:**
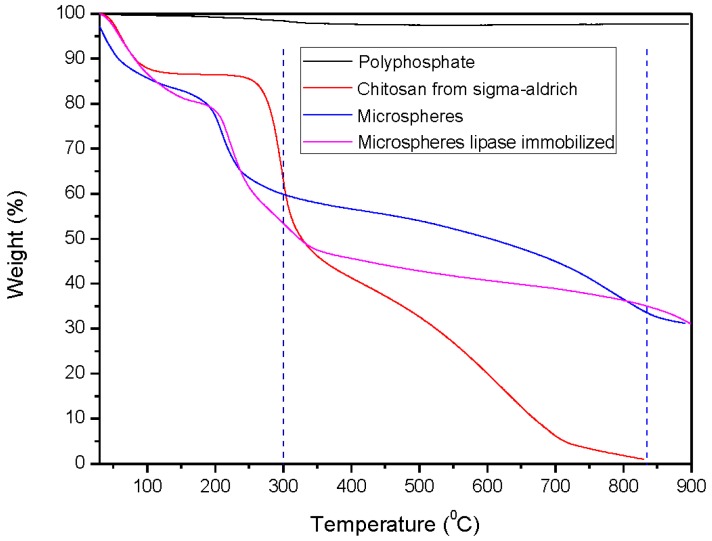
Thermogravimetric Analysis (TG) of chitosan, polyphosphate, microspheres and LPCS biocatalyst. Experiments have been performed with 10 mg of each sample, heating rate of 10 °C·min^−1^ in a synthetic air atmosphere. Other specifications are described in Section 3.

In the thermal decomposition curve of chitosan, two peaks were observed: the first one, which denoted an endothermic reaction, corresponded to the process of dehydration, and it was observed at approximately 263 °C; the second one, which denoted an exothermic reaction, corresponded to the process of chitosan decomposition, which continued beyond the temperature limit, and resulted in the formation of a residual solid mass of 1.8% (Figure 2).

Using polyphosphate, there was almost no decomposition of the sample, even at 900 °C, as it is a vitreous precursor. The thermal resistance of the microspheres was higher than that of the precursors because of the strong interactions between sodium polyphosphate and chitosan. Figure 2 also shows that the first decomposition event occurred between 150 °C and 250 °C, depending on the microspheres sample, and the other decomposition events were extended over a wide temperature range for the sample without lipase (called microspheres). When using the immobilized lipase sample, its thermal decomposition occurred between 299 °C and 834 °C.

The residual mass of chitosan was 1.8%, whereas the sample of sodium polyphosphate did not exhibit any loss in mass. On the other hand, the residual masses were 36.8% and 35% when the microspheres were used with or without lipase, respectively. This allowed concluding that virtually all of the residual mass was sodium polyphosphate.

#### 2.1.3. Confocal Raman Microscopy

**Figure 3:**
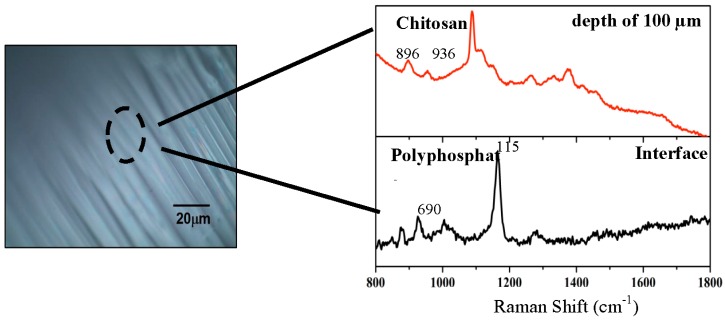
Confocal Raman spectra of two points located on the outside and inside of the microspheres, at a depth of 100 µm and intensity of 520.6 cm^−1^ band for a Si wafer. Other specifications are described in Section 3.

Confocal Raman microscopy suggested that the shells of the microspheres were composed of sodium polyphosphate, and the cores of the spheres were composed mainly of chitosan (Figure 3).

The peaks for polyphosphate, which were used as signature peaks, were of P-O-P vibrations and PO^2−^ stretches, which were observed at approximately 690 and 1157 cm^−1^, respectively [51]. Although the spectra of chitosan were similar to that of polyphosphate, it was possible to differentiate between them. The spectral profiles exhibited by the cores of the microspheres suggested that chitosan was the main component, because the peaks were observed at 896 and 936 cm^−1^, which were characteristic of C-H bending vibrations and C-N stretching [58].

The extent of the encapsulation through immobilization process of the lipase to form LPCS biocatalyst was next to 100%, and no protein was detected in the supernatant by the Bradford method, which suggested the complete encapsulation of the enzyme in the microspheres.

### 2.2. Characterization of LPCS

#### 2.2.1. Preservation of the Catalytic Activity of the Lipase by LPCS Immobilization

**Figure 4:**
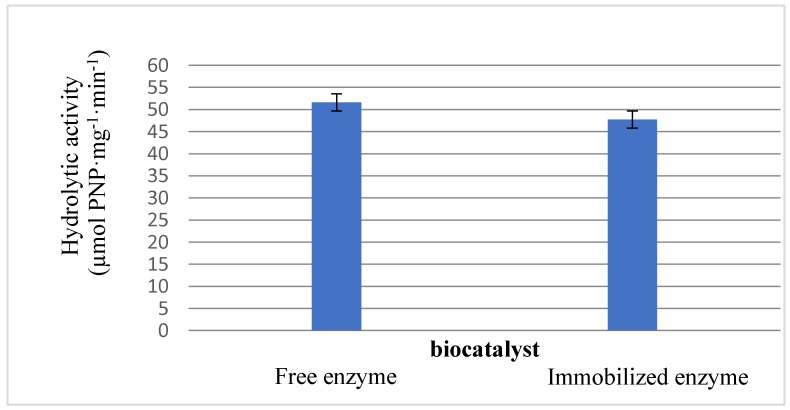
Hydrolytic enzyme activity was determined using 0.046 mL of free enzyme solution (6.5 mg/mL buffer pH 7) or 5 mg of dry microspheres, 3 mL of aqueous buffer at pH 7.0 and 25 °C and 0.3 mL of 20 mM PNPA in acetone under gently stirring. The enzyme was immobilized as described in the text. Other specifications are described in Section 3.

The ability of the biocatalyst to catalyze hydrolytic reactions was preserved upon its encapsulation inside the polyphosphate-chitosan support (Figure 4), which maintained 92% of the hydrolytic activity of the free enzyme. This proved that the support that was developed in this study did not compromise the catalytic activity of the enzyme [7].

#### 2.2.2. Effect of Reaction Temperature on the Activity of LPCS

**Figure 5:**
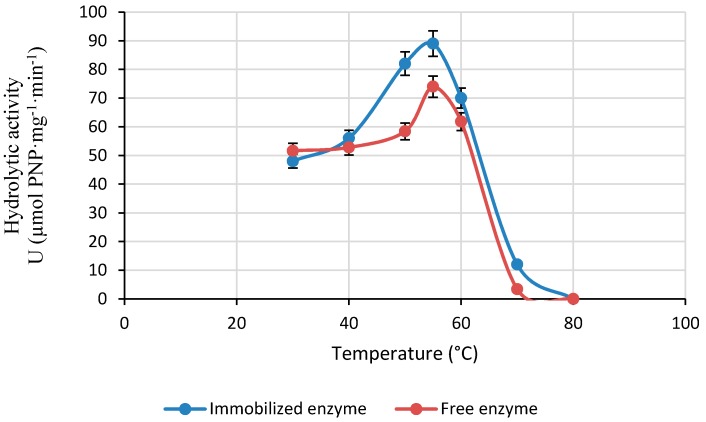
Effect of the temperature on the activity of different CALB preparations. Values expressed in Enzymatic Activity Unit (U), defined as the capacity of the catalyst (1 mg) to hydrolyze 1 µmol of PNP per minute. Experiments have been performed at pH 7.0 (3 mL, 0.05 M) using PNPA (300 µL, 20 mM) as substrate and 5 mg catalyst per 10 min at 30 °C. Other specifications are described in Section 3.

The hydrolytic activity of the immobilized enzyme was evaluated at different temperatures. Figure 5 shows that the activity of the immobilized enzyme increased up to 55 °C and decreased at temperatures higher than 60 °C; the enzyme became fully inactive at 80 °C while the free enzyme is fully inactive at 70 °C. The optimum temperature of the immobilized lipase was similar to other reports described in the literature [15].

#### 2.2.3. Catalytic Ability of the Enzyme Immobilized Using Different Solvents

**Figure 6:**
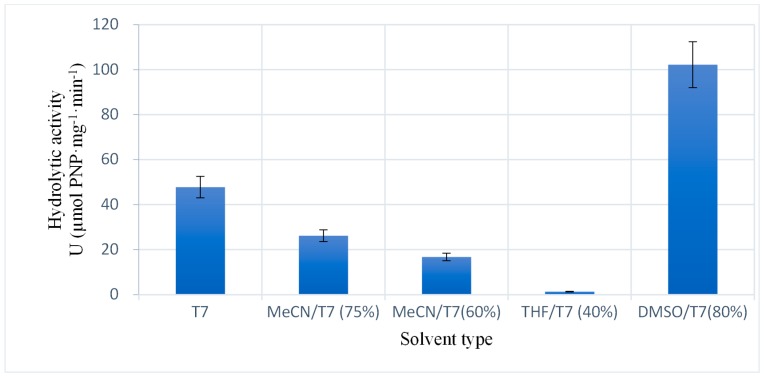
Effect of organic solvents on the enzyme activity on CALB immobilized in LPCS. Values are expressed in Enzymatic Activity Unit (U), defined as the capacity of the catalyst (1 mg) to hydrolyze 1 µmol of PNP per minute. Experiments have been performed at 30 °C using PNPA as substrate. MeCN: Acetonitrile; T7: buffer at pH 7; THF: tetrahydrofuran, DSMO, dimethyl sulfoxide. Other specifications are described in Section 3.

The hydrolytic activity of the enzyme immobilized in the LPCS support was studied using different mixtures of solvents and aqueous buffers (Figure 6). LPCS exhibited better performance in the medium composed of dimethyl sulfoxide/phosphate buffer 7.0 mixture; the enzyme activity in this medium was more than double compared to the one detected in a pure aqueous medium contradicting results presented by Bommarius, that showed a deactivation effect of DMSO [59]. This suggests the good properties of the new biocatalyst. The lowest activity was obtained when tetrahydrofuran/buffer 7.0 was used as the solvent for the reaction, as shown in Figure 6.

Acetonitrile had a greater negative effect at 60% than at 75%. It is important to note that although the hydrolytic activity of the enzyme in the different solvents was different, the immobilized enzyme was reasonably active in all of them, with the exception of the THF solvent where the enzyme had negligible activity. It was also possible to highlight that the use of binary solvent mixtures with higher dielectric constant (water, acetonitrile, and DMSO) resulted in higher hydrolytic activity of the enzyme. The high resistance of the enzyme to negative effects of organic solvents, together with the prevention of enzyme aggregation, could also be partially due to the partition of the organic solvents away from the highly hydrophilic environment of LPCS. This phenomenon of solvent partition has been described in other cases using other ionic polymers [60,61,62].

#### 2.2.4. Stability of the Enzyme Immobilized in the LPCS Support at Different Values of pH

**Figure 7:**
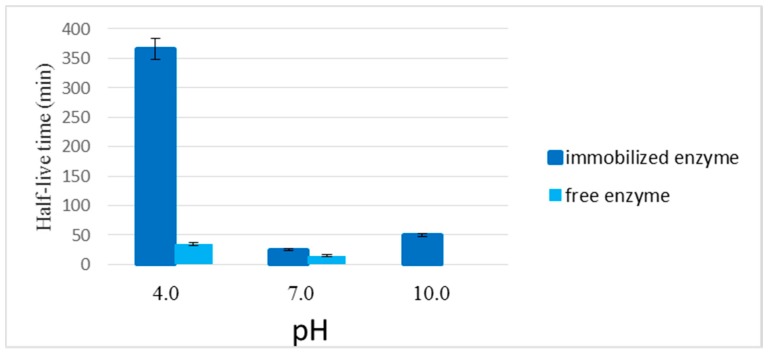
Half-lives (min) of CALB immobilized in different pH (4.0, 7.0 and 10). Experiments have been performed at 65 °C following the activity with PNPA as described in Section 3.

The analysis of the stability of LPCS at different pH values and 65 °C is represented in Figure 7. The temperature of 65 °C for the pH test was selected in order to render the least system stable to be able to detect some enzyme inactivation after a reasonable time-period.

The enzyme was highly stable at pH 4.0, with a half-life of 366 min. When the medium was basic (pH 10.0), the biocatalyst half-life was reduced to 50 min, and even lower, to 25 min at pH 7.0. At pH 7, the enzyme could interact with both cationic and anionic groups in the support, which could be the driving force for the enzyme inactivation being faster at neutral pH value than at extreme pH values.

These results suggested greater thermal stability of the enzyme immobilized in the LPCS at different values of pH than those obtained when the enzyme was immobilized in a pre-formed chitosan/polyphosphate support via ion exchange [63]. Comparatively Badgujar and Bhanage tested three enzyme biocatalysts, being the best one that containing chitosan in the structure, but the enzyme activity was reduced 45 times [64], while by the novel method proposed in this paper, more than 90% of the enzyme activity was maintained after immobilization. The strong ion exchange between the enzyme and the *ex novo* support, which involved the entire enzyme structure and reduced enzyme mobility, may be the reasons for the excellent results in terms of activity and stability [65].

The immobilized enzyme stability in acidic media agreed with the results of the confocal Raman microscopy which suggested that polyphosphate constituted the shell of the microspheres, because this polymer was more resistant in acidic media than chitosan, which was highly soluble in acidic media. In this way, under acidic pH, the microspheres appear to be completely stable, and this prevented enzyme inactivation.

Moreover, this methodology presents potential in the application of multimeric enzymes immobilization because low pH (acidic pH) values may promote the subunit dissociation of many multimeric enzymes and, consequently, the inactivation of enzymes [26] The dissociation will be prevented if all enzyme subunits are trapped [26].

#### 2.2.5. Enzymatic Synthesis Catalyzed by LPCS of the Compound Responsible for the Aroma of Jasmine

**Figure 8:**
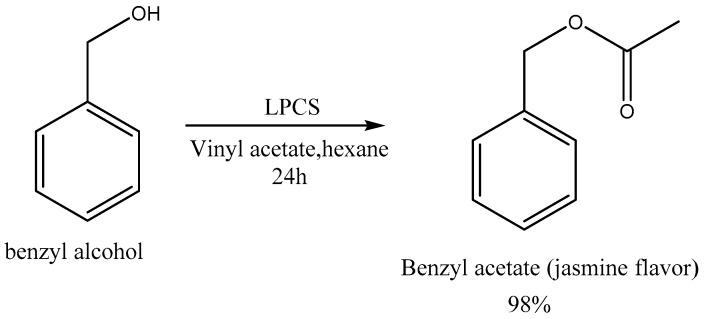
Scheme of the enzymatic production of the aroma of jasmine (benzyl acetate). Other specifications are described in Section 3.

**Figure 9:**
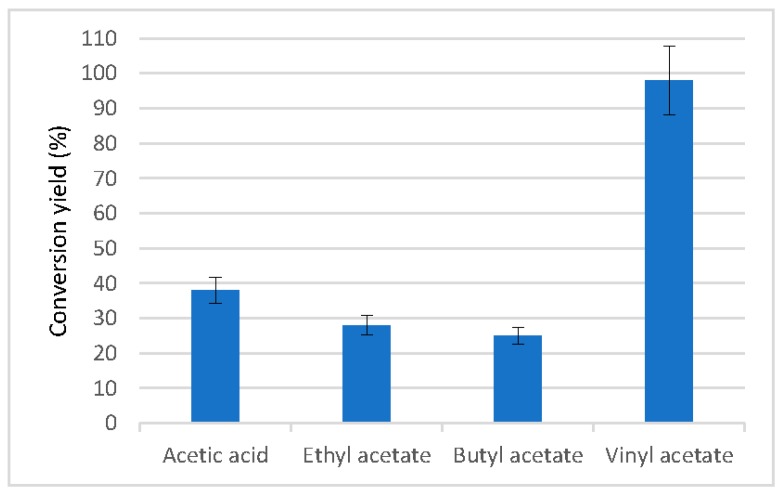
Effect of acyl donors on the acetylation of benzyl alcohol catalyzed by CALB immobilized in LPCS support. Experiments have been performed at 30 °C, using 5 mg of dry microspheres, 0.02 mL of benzyl alcohol, 0.05 mL of different acyl donors completing the volume with hexane to 2 mL. The reaction was left to proceed for 24 h. Other specifications are described in Section 3.

Another potential application of LPCS, which was evaluated in this study, was its ability to artificially produce the compound responsible for the aroma of jasmine (Figure 8). This compound has wide applications in the cosmetic and perfume industries [66]. The immobilized enzyme was able to catalyze the acetylation of benzyl alcohol with different acyl donors. The activated acyl donor that permitted the highest enzyme activity in the acetylation reaction was vinyl acetate, which gave also the highest maximum yield. When acetic acid (this was a direct esterification reaction), ethyl acetate, or butyl acetate (these were transesterification reactions) were used, the conversion levels were below 40% (Figure 9).

**Figure 10:**
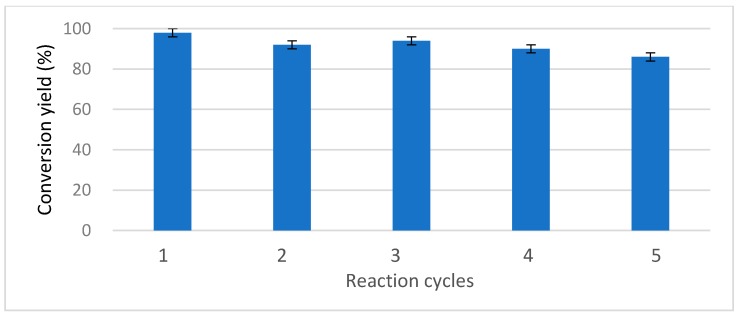
Operational stability of CALB immobilized in LPCS during the production of benzyl acetate. Experiments have been performed at 30 °C, using 5 mg of dried microspheres, 0.02 mL of benzyl alcohol, 0.05 mL of acyl donor (vinyl acetate) completing 2 mL with hexane, reaction time of 24 h. Then the biocatalyst was washed with hexane, dried at 30 °C for 20 min and finally submitted to a new reaction cycle. Other specifications are described in Section 3.

The yields of the direct esterification reaction were higher than those of the transesterification reactions using esters, because in the latter, the released alcohols might compete with benzyl alcohol reducing the final yields. In any case, in the reaction involving vinyl acetate, during which no such competitive alcohol was released, a high yield was observed within a short time (74% conversion after 12 h, and 98% after 24 h (Figure 10)). These results were better than those previously reported on the synthesis of esters used in perfumes. For example, conversion levels of less than 90% were reported after 24 h of reaction when the commercial enzyme Lipozyme RMIM was used using supercritical carbon dioxide as solvent, that is, a reactive medium more technologically complex than the one presented in this work [66].

Conversion yields of 97.3% in the synthesis of these esters with jasmine aroma were obtained by enzymatic catalysis using commercial enzymes as biocatalysts. In those examples Lipozyme TL IM (lipase from *Thermomyces lanuginosus* immobilized on silica gel, 0.25 U/mg) and Novozym 435 (lipase B from *Candida antarctica* immobilized on macroporous resin, 10 U/mg) were used to esterify benzyl alcohol for 24 h. These conditions are similar to those used in this work and they are also reported in the literature [67] and the comparative among the results suggest the promising LPCS biocatalyst performance in organic synthesis.

In another study, the enzymatic syntheses of methyl butyrate and octyl acetate (important esters for the industrial fragrances) were carried out, using commercial immobilized lipase from *Rhizopus oryzae* (NRRL 3562) and performing the transesterification reaction under solvent-free conditions. Molar conversions of 70.42% and 92.35% were obtained in reaction times of 14 and 12 h for methyl butyrate and octyl acetate, respectively [68].

This enzymatic process has a great interest, both from environmental and operational perspectives, because in typical chemical acetylation reactions, toxic catalysts (pyridine) are often used with onerous recovery methods, and in general, they are not ecologically sustainable [69,70].

#### 2.2.6. LPCS Recycling

In order to evaluate the possibility of the reutilization of LPCS in this reaction, the same LPCS biocatalyst was utilized for five cycles. It was possible to observe that the reaction yields that were obtained using LPCS exhibited a progressive but relatively small decrease over successive cycles, from 98% to 86% after the fifth cycle (Figure 10).

**Figure 11:**
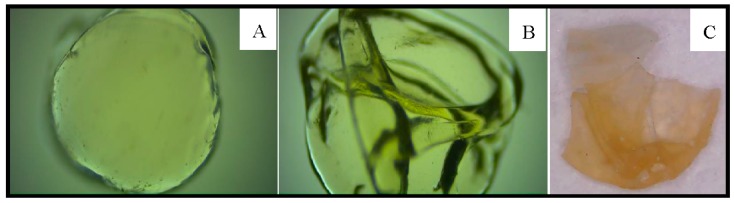
Photomicrographs of the microspheres at 50× magnification factor. (**A**) microsphere just after being produced; (**B**) microspheres after 30 days of storage under wet conditions at room temperature; (**C**) microspheres after 24 h of being submitted to heating at 30 °C in a vacuum dryer. Other specifications are described in Section 3.

The microspheres lost their structures when they were stored under wet conditions at room temperature after 30 days. This could be clearly visualized by the release of the liquid that was trapped inside the LPCS biocatalyst into the medium (as shown in Figure 11). However, when dried, the spheres retained their shape, the particles became harder, and the enzyme remained immobilized and active within them during after 30 days. This implied that the use of LPCS in an anhydrous medium would be of great interest.

## 3. Materials and Methods

All experiments were performed at a minimum in duplicate.

### 3.1. Materials

The enzyme that was used in this study was lipase B from *Candida antarctica* (CALB), which was supplied by Novozymes (Alcobendas, Spain). Chitosan, benzyl alcohol and *p*-nitrophenyl acetate (PNPA) were obtained from Sigma-Aldrich (Saint Louis, MO, USA), sodium polyphosphate was supplied by Merck (Kenilworth, NJ, USA) and used without further refinement. Deionized water was used in this study.

### 3.2. Equipments Used for the Characterization of the Biocatalyst

Fourier transform infrared (FTIR) spectra from 4000 to 400 cm^−1^ were recorded using an FTLA 2000-102 FTIR spectrophotometer (ABB–Bomem, Zurich, Switzerland) using the KBr pellet technique at room temperature.

Thermogravimetric (TG) analysis was performed using a thermogravimetric analyzer (model TGA/SDTA851e; Mettler-Toledo, Columbus, OH, USA) by heating the samples from 30 °C to 900 °C at 10 °C·min^−1^ in a synthetic air atmosphere.

The Raman spectra were obtained using an inVia Qontor Raman microscope (Renishaw, Gloucestershire, UK) coupled to a Leica DM2500 M microscope (Leica, Mannheim, Germany), a Renishaw MS 20 motor stage with axial and lateral resolution of 0.10 mm, which was controlled using the wire 3.6 software (Renishaw, Gloucestershire, UK), excited with radiation at 632.8 nm (He-Ne, Renishaw) and at 785 nm (diode, Renishaw). The scattered radiation was dispersed using a diffraction grating of 1200 lines·mm^−1^, and recorded using a Peltier-cooled CCD camera. The samples were observed using a LEICA objective lens with a numerical aperture of 0.9 and magnification of 50×.

A QP2010SE Plus GC/MS apparatus (Shimadzu, Kyoto, Japan) using a 30-m Rtx^®^-5MS (95% dimethylpolysiloxane and 5% diphenyl) capillary column, with an internal diameter of 0.25 mm and film thickness of 0.1 μm of the fixed phase was used; The temperatures of the injector and detector were 240 °C and 280 °C, respectively. The column conditions were: 60–80 °C at 5 °C·min^−1^, held for 3 min, then, from 80 °C to 280 °C at 30 °C·min^−1^, remaining at this temperature for 10 min using He as the gas for entrainment with a flow rate of 1.0 mL·min^−1^. Analysis was performed using the mass spectrometer in scan mode with an analysis time in 23.67 min; The mass spectra were recorded in the range of 35 to 500 daltons per electron impact (EMIE) with an ionization energy of 70 eV (voltage of 1.5 KV) using a quadrupole mass analyzer and an ion source at 240 °C.

The enzyme activities of free lipase and immobilized lipase were monitored by UV–Vis spectroscopy using a FEMTO 600 S spectrophotometer (FEMTO Indústria e Comércio de Instrumentos, Sao Paulo, Brazil) at a specific wavelength.

Images of the microspheres were observed using a LABOMED/ANALITICA LX 400 optical microscope (LABOMED, Los Angeles, CA, USA) equipped with Siedentopf binocular and trinocular heads inclined at 30°, and 360°, respectively rotation an interpupillary distance of 48–75 mm, with 10×/20 mm wide-field eye-pieces of optical Infinite RP Series, and Achromatic Flat DIN Objectives of 4× (WD 30.0 mm), 10× (WD 7.0 mm), 40× (WD 0.65 mm; retractable), and 100× (WD 0.23 mm).

### 3.3. Synthesis of the Microspheres from Chitosan and Polyphosphate

Microspheres were synthesized by adding a 75% to 85% deacylated chitosan (CH, 2% *w*/*v*) solution dropwise to a solution of sodium polyphosphate (PP), (Merck ≥68%, 0.2 M), with agitation at room temperature. Microsphere samples with a molar ratio (PP:CH) ranging from 10:1 to 1000:1 were synthesized.

**Figure 12:**
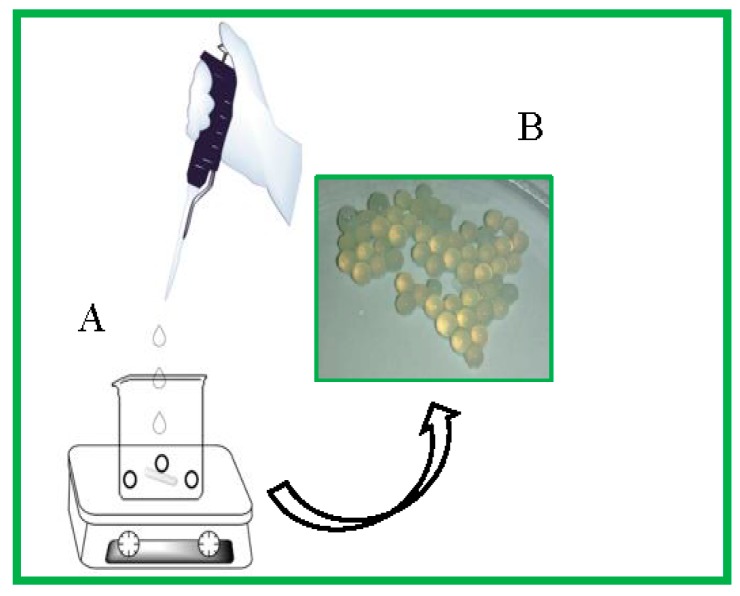
Schematic representation of the procedure for the formation of microspheres, in (**A**) CALB solution (0.38 mL; 1 mg/mL) dissolved in 2.5 mL of 2% (*w*/*v*) chitosan solution was added dropwise to 10 mL of 0.2 M sodium polyphosphate and (**B**) the image of microspheres. Other specifications are described in Section 3.

The chitosan solution was added dropwise to an aqueous solution of sodium polyphosphate at a concentration of >0.2 M. Ten milliliters of each of the polymer solutions was used; then, the produced spheres were recovered by decantation, and washed with distilled water (Figure 12). The standard molar ratio of the two polyelectrolytes (polyphosphate:chitosan) used was 1000:1, since this ratio resulted in the synthesis of microspheres with higher mechanical resistance than those generated at the ratios of 100:1 and 10:1. The supernatants were collected for further analysis by UV-Vis spectroscopy.

### 3.4. Immobilization of CALB

CALB solution (0.38 mL; 1 mg/mL) was mixed with 2.5 mL of 2% (*w*/*v*) chitosan solution, and the system was subjected to magnetic stirring for 30 min; then, all the mixture of chitosan and CALB was added dropwise to 10 mL of sodium polyphosphate solution, as described in the previous section, and subjected to 30 min of mild magnetic stirring. The formation of microspheres was instantaneous for each added drop. The new biocatalyst-polyphosphate-chitosan support that encapsulated the lipase was referred to as LPCS.

### 3.5. Determination of the Concentration of the Immobilized Enzyme

The concentration of the immobilized enzyme in the support was determined according to Equation (1):

(1) Amount of immobilized enzyme, (%)=100×(1−protein in supernatant/initial ofered protein)

### 3.6. Hydrolytic Activity

The evaluation of the hydrolytic activity of CALB was based on the hydrolysis of PNPA. An aliquot of 300 µL of 20 mM PNPA in was added to 3 mL of 0.05 M phosphate buffer solution pH 7.0. Then, 5 mg of the immobilized enzyme was added to the system with stirring (175 rpm) for 10 min. After this, the solution was filtered, and the concentration of PNP that was released was quantified using a spectrophotometer at 410 nm. One unit of hydrolytic activity (A) was defined as the amount of enzyme required to release 1 µmol of PNP per minute (µmol·min^−1^). At certain instances, the temperature was varied between 30 °C and 80 °C, and different solvents were added. The hydrolytic activity of the enzyme was calculated according to Equation (2):

(2) $$\mathrm{Hydrolytic}\;\mathrm{activity}\;\left(A\right)=\frac{\mathrm{Amount}\;\mathrm{of}\;\mathrm{PNP}\;\left(\mu \mathrm{mol}\right)}{\mathrm{Amount}\;\mathrm{of}\;\mathrm{immobilized}\;\mathrm{enzyme}\;\left(\mathrm{mg}\right)\times \mathrm{time}\;\left(\min\right)}$$

### 3.7. Activity in Organic Solvents

The activity of the enzyme that was encapsulated in the microspheres was tested in different mixtures of organic solvents (acetonitrile (MeCN); tetrahydrofuran (THF) and dimethyl sulfoxide (DMSO) and phosphate buffer pH 7.0 (T7)), according to the following methodology: 3.0 mL of the evaluated solvent was added to 300 µL of 20 mM PNPA in acetone with 5 mg of dried immobilized enzyme.

### 3.8. Effect of pH

For the analysis of the thermal inactivation of the biocatalyst under stress conditions, 5 mg of biocatalyst was suspended in 1 mL of 10 mM sodium acetate at pH 4, sodium phosphate at pH 7, or sodium carbonate at pH 9, in all cases at 65 °C (±2). Periodically, samples were withdrawn, and the activity was measured using PNPA. The deactivation constant and half-life of each immobilized derivative were calculated according to the model of Sadana and Henley [71] using Microcal Origin version 8.1 (Microcal Software, Inc., Malvern, UK) [44].

### 3.9. Enzymatic Synthesis of Esters—Effect of the Acyl Donor Groups and Enzymatic Synthesis of the Compound Responsible for the Aroma of Jasmine

A quantity of 5 mg of the immobilized enzyme was added to 20 µL of benzyl alcohol and 50 µL of acyl donor (vinyl acetate, acetic acid, ethyl acetate, and butyl acetate) in 2 mL of hexane solution. Then, aliquots were withdrawn after 12 and 24 h, and analyzed by gas chromatography coupled with mass spectrometry using chiral columns.

### 3.10. Reuse of the Catalyst

For analyzing the reusability of the immobilized enzyme, a reaction with 20 µL of benzyl alcohol was performed. The quantities of (5 mg) the immobilized enzyme and vinyl acetate (50 µL) were maintained with hexane (2 mL) as the solvent. The reaction products were analyzed at different reaction times (12 and 24 h). After 24 h, the reaction medium was filtered, and the immobilized enzyme was separated from the reaction medium by filtration. Finally, the support was washed twice with 5 mL of hexane and was dried at room temperature for 30 min, and then, subjected to a new reaction cycle (five times).

## 4. Conclusions

The new proposed LPCS enzymatic biocatalyst, where the enzyme is trapped by oppositely charged polyions, sodium polyphosphate and chitosan, has proved to be an efficient protocol for the physical entrapment of enzymes (that is, microencapsulation). The prepared biocatalyst allowed its application in organic media, with potential use in organic synthesis.

Results showed that the immobilized lipase presented high thermal stabilization and, maintained high activity in organic solvents, and, finally, the biocatalyst stability in extreme pH values (pH 4 and 10) is remarkable. The stability presented at pH 4 was higher than the values reported in the literature for other immobilized CALB preparations under these conditions, with a half-life of 366 min at 65 °C. The biocatalyst showed good performance for the reactions of enzymatic synthesis of the compound responsible for the aroma of jasmine with a yield of 98% after 24 h of reaction.
